# The Immune Microenvironment in Multiple Myeloma: Friend or Foe?

**DOI:** 10.3390/cancers13040625

**Published:** 2021-02-05

**Authors:** Raquel Lopes, Joana Caetano, Bruna Ferreira, Filipa Barahona, Emilie Arnault Carneiro, Cristina João

**Affiliations:** 1 Lymphoma and Myeloma Research Programme, Champalimaud Centre for the Unknown, 1400-038 Lisbon, Portugal; raquel.lopes@research.fchampalimaud.org (R.L.); joana.caetano@research.fchampalimaud.org (J.C.); bruna.ferreira@research.fchampalimaud.org (B.F.); filipa.barahona@research.fchampalimaud.org (F.B.); emilie.carneiro@research.fchampalimaud.org (E.A.C.); 2 Faculty of Medicine, University of Lisbon, 1649-028 Lisbon, Portugal; 3 Hemato-Oncology Department, Champalimaud Centre for the Unknown, 1400-038 Lisbon, Portugal; 4 Faculty of Medical Sciences, NOVA Medical School, 1169-056 Lisbon, Portugal

**Keywords:** multiple myeloma, tumor microenvironment, cancer immunity, immunotherapy

## Abstract

**Simple Summary:**

The crosstalk between multiple myeloma and immune cells within the bone marrow niche has been identified as an emerging hallmark of this hematological disease. As our knowledge on this interplay increases, it becomes more evident that successful treatment approaches need to boost the body’s natural defenses through immunotherapy. The present review will focus on the mechanisms by which myeloma cancer cells turn immune populations into their “partners in crime”. Additionally, we will provide an overview of currently ongoing pre-clinical studies targeting the bone marrow immune microenvironment.

**Abstract:**

Multiple myeloma (MM) is one of the most prevalent hematological cancers worldwide, characterized by the clonal expansion of neoplastic plasma cells in the bone marrow (BM). A combination of factors is implicated in disease progression, including BM immune microenvironment changes. Increasing evidence suggests that the disruption of immunological processes responsible for myeloma control ultimately leads to the escape from immune surveillance and resistance to immune effector function, resulting in an active form of myeloma. In fact, one of the hallmarks of MM is the development of a permissive BM milieu that provides a growth advantage to the malignant cells. Consequently, a better understanding of how myeloma cells interact with the BM niche compartments and disrupt the immune homeostasis is of utmost importance to develop more effective treatments. This review focuses on the most up-to-date knowledge regarding microenvironment-related mechanisms behind MM immune evasion and suppression, as well as promising molecules that are currently under pre-clinical tests targeting immune populations.

## 1. Introduction

Multiple myeloma (MM) is the second most frequent hematological cancer worldwide with a global incidence of 159,985 new cases in 2018 [1]. MM is an aggressive malignancy characterized by the clonal expansion of terminally differentiated B cells in the bone marrow (BM) and clinically defined by increased BM plasmacytosis, serum and/or urine monoclonal immunoglobulin, secretion of free light chains, hypercalcemia, renal insufficiency, anemia, and bone pain due to osteolytic disease [2,3]. This disease is preceded by an asymptomatic premalignant condition termed monoclonal gammopathy of uncertain significance (MGUS), and in some patients, it is possible to distinguish an intermediate stage called smoldering MM (SMM) [4,5].

The MM BM niche is a heterogeneous system where the crosstalk between neoplastic plasma cells and immune populations, from both myeloid and lymphoid lineage, plays a critical role in myelomagenesis and maintenance. Indeed, myeloma cells display a wide variety of mechanisms that induce a permissive microenvironment, allowing immune evasion and favoring its own proliferation, survival, migration, and drug resistance [6,7]. Consequently, the search for novel and more effective drugs that kill tumor cells and/or boost the immune system has increased in the last years. Yet, despite substantial progress in classification, staging, and treatment, the majority of MM patients still have only a median of four to five years of survival after starting treatment [8]. Many of these patients relapse due to the presence of residual malignant cells that may be undetectable by currently available monitoring techniques as next generation sequencing or flow cytometry [9]. A major scientific and clinical challenge in this disease is finding a balance between myeloma cell killing efficacy and toxicity for patients. Therefore, considering the role of immunity in MM, a deeper understanding of the interaction between neoplastic plasma cells and the BM immunome is of utmost importance for the development of more effective and safe treatments.

Of note, there are other non-immune populations, such as mesenchymal stromal cells or osteoclasts/osteoblasts, as well as metabolic pathways that are deregulated, leading to drug resistance and myeloma’s escape from immune surveillance [10,11,12]. Notwithstanding, this review exclusively focuses on the current state of the art regarding the BM immune microenvironment.

## 2. Myeloma Immune Microenvironment: From Surveillance to Immune Escape—The Concept of Immunoediting

MM is an excellent cancer immunoediting example in which a premalignant equilibrium phase—MGUS—anticipates the escape phase of active disease [13,14]. The precursor stages, as MGUS and SMM, already harbor malignant clones characterized by genomic changes (e.g., IgH translocations or hyperdiploidy) [15,16]. MM progression is a process far more complex than the first alterations occurring in these neoplastic cells. This evolution is driven by subsequent acquisition of other genetic transformations, including MYC translocations, 1q gains, or TP53 mutations [17,18]. Presently, it is evident that composition and function of the immune components in the BM myeloma niche relate with MM progression and aggressiveness [19,20,21,22].

**Figure 1:**
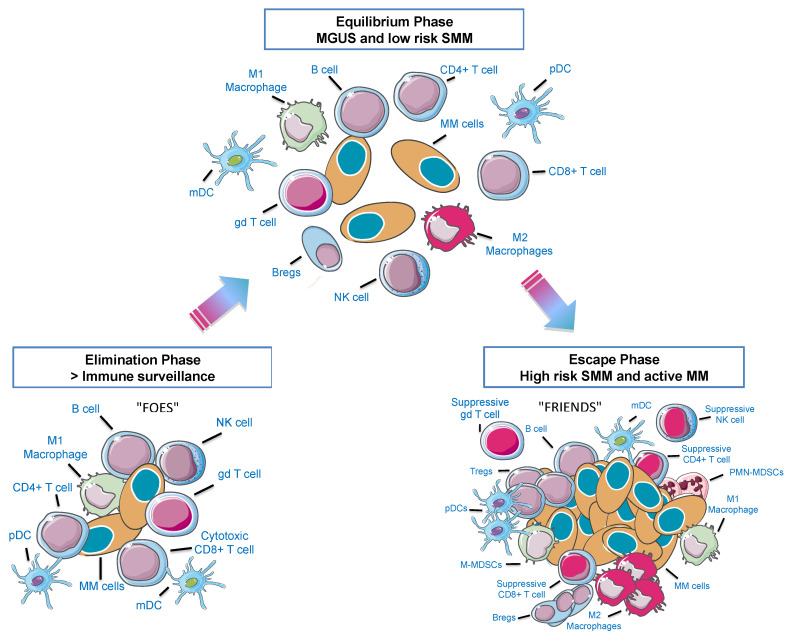
The 3E’s of immunoediting in the multiple myeloma (MM) bone marrow (BM) microenvironment. Immune cells can either eliminate cancer cells or facilitate escape from immune surveillance according to environmental cues. When neoplastic plasma cells start to arise, they can be recognized as strange and be eliminated by immune cells (Elimination Phase—“FOES”). However, cells can reach an equilibrium phase that involves a continuous eradication of myeloma cells. At the same time there is a Darwin selection of the most resistant clones and a decrease of immunogenic tumor cells. This stage is probably the longest phase and may occur over a period of several years, as it happens in monoclonal gammopathy of uncertain significance (MGUS) and low risk smoldering MM (SMM) patients. Afterwards, this novel population of myeloma clones that can model immunity, will grow and expand (Escape Phase—“FRIENDS”). pDC: plasmacytoid dendritic cell; mDC: myeloid dendritic cell, gd: gamma-delta T cells; Bregs: regulatory B cells; Tregs: regulatory T cells; M-MDSCs: monocytic myeloid-derived suppressor cells; PMN-MDSCs: polymorphonuclear MDSCs. Adapted from Dunn et al., 2004 [13].

A summary of the different phases of MM development and their crosstalk with the immune microenvironment are shown in Figure 1. Cell populations relevant in the MM immune niche will be addressed subsequently.

### 2.1. Myeloid-Derived Suppressor Cells

In the last years, myeloid-derived suppressor cells (MDSCs), a heterogeneous population of immature myeloid cells, have emerged as negative regulators in infection, autoimmune diseases, sepsis, and cancer [23,24,25]. On the basis of their morphological and phenotypic properties, these cells can be further divided into polymorphonuclear MDSCs (PMN-MDSCs) and monocytic MDSCs (M-MDSCs) [26]. Functionally, it is known that PMN-MDSCs are more suppressive than M-MDSCs [27]. 

The role of MDSCs as MM-promoters in the BM microenvironment is just starting to be unraveled. MDSCs accumulate in the BM of both patients and MM-bearing mice [28,29,30,31,32] and their levels correlate with the disease stage and prognosis [30,33]. Interestingly, a study from Binsfeld et al. showed that reduced levels of PMN-MDSCs were associated with less formation of blood vessels, and M-MDSCs were identified as osteoclast precursors, favoring disease progression [27]. These results suggest that MDSCs sub-populations act in different ways in the BM milieu of MM. Thus, we could hypothesize that targeting MDSCs in general could not only improve anti-MM immunity, but also modulate angiogenesis and osteolytic bone disease in MM patients.

The S100A9 knock-out (KO) murine model represents an excellent example to study the effect of MDSCs depletion, since they are deficient in their ability to accumulate MDSCs [34]. Therefore, several pre-clinical studies have used this model to study the involvement of these cells in the promotion of an impaired BM immunity in MM. For instance, Ramachandran and colleagues showed that upon tumor establishment, MDSCs accumulate in the BM of MM-bearing mice, and this was accompanied by the inhibition of anti-tumor cytotoxicity and a decrease in the presence of Th1 CD4+T cells. By using the S100A9 KO murine model, they found a reduced accumulation of MDSCs and a re-establishment of anti-tumor immunity, decelerating MM progression [28]. Additionally, Görgün and colleagues showed that neoplastic plasma cells and MDSCs interact in a “win-win” mode within the BM niche. While myeloma cells promote MDSCs development and survival, MDSCs create a protective environment by inducing NK T and CD8+T cells anergy. This effect is mediated by soluble factors produced by MDSCs, such as inducible nitric oxide synthase (iNOS), arginase (ARG1), reactive oxygen species (ROS), and immunosuppressive cytokines, including IL-6 and IL-10. This study also demonstrated that the expression of these cytokines was abrogated using currently available therapies, such as bortezomib and lenalidomide. However, the number or functional immunosuppression of MDSCs was not abolished by in vitro exposure to these agents [30]. Conversely, in another study by Wang et al., treatment with these same agents resulted in a decreased frequency of MDSCs [33]. The data on the effect of currently available therapies tackling MM MDSCs are still inconsistent and need further study. Nonetheless, Ramachandran and colleagues showed that these suppressive effects can be reduced by decreasing the number of MDSCs, leading to a significant delay in myeloma proliferation and improved response to chemotherapy (e.g., doxorubicin) [35]. Thus, these results suggest that one effective way to overcome chemoresistance in MM might be through the combination of currently available drugs with MDSCs-depleting agents. Also aligned with this hypothesis, Wang et al. used the murine 5T33MM model to demonstrate an indirect mechanism supporting MM progression, in which MDSCs uptake BMSC-derived exosomes in the BM microenvironment. This phenomenon induces MDSCs expansion in vitro and enhances the immunosuppression on T cells through the secretion of nitric oxide. Specifically, these exosomes can improve MDSCs survival, not only by activating STAT3 and STAT1 pathways but also by increasing anti-apoptotic proteins (e.g., Bcl-xL and Mcl-1) [36].

Taken together, these studies demonstrate that MDSCs work in favor of myeloma plasma cells and are able to regulate the efficacy of currently used chemotherapies and novel immunotherapeutic agents [37,38]. Therefore, MDSCs-depleting/targeted drug strategies may relieve immunosuppression found in the BM MM niche and subsequently augment the anti-tumor effect of concomitant therapies for MM patients.

### 2.2. Macrophages

Macrophages are professional phagocytic cells that play a crucial role in pathogen elimination as well as in tissue repair. They are classified under two major phenotypes: M1 and M2. In a tumor bed, M1 pro-inflammatory macrophages act as potent anti-tumor effectors by antagonizing the suppressive activities of pro-tumoral cells, whereas anti-inflammatory M2 macrophages are involved in anti-tumor suppression [39,40]. During neoplastic progression, M1 macrophages may gradually lose their anti-tumor properties and acquire a M2-like phenotype, also known as Tumor-Associated Macrophages (TAMs). The role of TAMs in cancer development have been extensively studied, showing their potential value not only as diagnostic and prognostic biomarkers, but also as therapeutic targets [41,42].

In MM, several studies reported a correlation between a higher CD163+ macrophages infiltration of the BM and a more aggressive disease, shorter patient survival, and response to treatment [43,44,45]. In addition, a recent publication revealed that higher levels of CD68+/CCR2+ macrophages in patients treated with bortezomib were correlated with a worse outcome, suggesting that pro-inflammatory macrophages represent a potential predictive biomarker for MM resistance to bortezomib therapy [46].

Currently explored MM therapeutic strategies involve macrophage recruitment inhibition to the tumor microenvironment or macrophage depletion by targeted apoptosis induction. For instance, a report by Beider et al. showed that myeloma-derived CXCL12 could attract and recruit monocytes. Of note, this migration could be further enhanced either by BM stromal cells alone, that also secrete CXCL12, or through their reciprocal interaction with neoplastic plasma cells, leading to monocytes’ differentiation towards a M2 phenotype. Moreover, researchers found that the CXCR4/CXCL12 axis was critically involved in these migratory signals, since the neutralization of CXCR4 abrogated monocyte recruitment. Finally, this immunosuppressive phenotype was shown to suppress T cell responses, promote the formation of novel blood vessels, support myeloma cell growth and chemoresistance [47]. Overall, this study supports that the presence of macrophages with an anti-inflammatory phenotype in the BM microenvironment may negatively affect prognosis and/or treatment response.

As macrophages keep an inherent anti-tumor potential in the BM niche, another promising approach involves macrophage reprograming. Injection of xenograft tumor mouse models with a combination of GM-CSF (Pro-M1) and 4-IPP (M2 inhibitor) was shown to induce reprogramming towards an M1 profile, at both gene and protein expression levels, as well as remarkable tumoricidal effects [48]. 

Altogether, these results demonstrate that macrophages are critical actors in the settlement and the progression of MM. Moreover, macrophages can be “manipulated” by myeloma cells to serve the cause of the tumor. Notwithstanding, several macrophages-reprogramming therapies are currently under study in order to recover them as partners instead of enemies.

### 2.3. Dendritic Cells

Dendritic cells (DCs), as professional antigen-presenting cells, act as a link between innate and adaptive immunity [49]. DCs can be further divided into two subclasses on the basis of their morphology and function: myeloid DCs (mDCs) and plasmacytoid DCs (pDCs) [50]. mDCs secrete IL-12 and play an important role in enhancing adaptive immune responses [51]. Conversely, pDCs are specialized IFN-producing cells, with an important role in the activation of Toll-like receptors (TLRs) such as TLR7 and TLR9 upon viral infections [52]. 

Although there is not a complete agreement in the literature regarding DCs frequency, phenotype, and function in MM patients’ BM versus healthy BM, it is assumed that MM patients’ BM DCs are functionally defective. Indeed, several research groups already demonstrated that some immunological properties of DCs are compromised during myelomagenesis, diminishing effective anti-tumor immune responses and leading to myeloma escape. This includes a lack of expression of HLA-DR, CD40, CD80, and CD86 molecules and defective antigen presentation compared to healthy samples [53]. Furthermore, some activation markers have been reported to be associated with the defective differentiation of monocyte-derived DCs (Mo-DCs) found in the peripheral blood (PB) of MM patients. For instance, in a study performed by Wang et al., higher production of IL-6 was found, as well as increased levels of p38 and STAT3, with the inhibition with Raf/MEK/ERK signaling pathways in MM-derived progenitor cells compared to healthy donors. Furthermore, treating these cells with an anti-IL-6 alone or with a p38 inhibitor not only increased the number of circulating DC but also restored their phenotype and functionality [54]. In another study from Kukreja et al., the crosstalk between DCs and neoplastic plasma cells was reported as supporting myeloma’s proliferation by RANK-RANK ligand and APRIL interactions, which can be abrogated through its blockade. In this regard, direct targeting of DCs may also be a fruitful treatment for MM. As such, in vitro blockade of RANKL and APRIL pathways have shown to inhibit DCs-mediated myeloma proliferation [55]. More recently, Shinde and colleagues demonstrated that Mo-DCs were functionally defective due to lower expression of IL12p70 and higher levels of IL-10 production in myeloma patients, leading to impaired T helper (Th) 1 response. Additionally, these cells displayed compromised CCR7-dependent migration to lymph nodes, which could be attributed to the higher production of IL-6 and activation of the p38 MAPK pathway found in samples from MM patients compared to healthy subjects [56]. Altogether, these data are particularly relevant because Mo-DCs are being considered to be used as vaccines, and it raises the question of whether they are the ideal vehicles for antigen delivery in MM treatment.

Furthermore, immature DCs were found to contribute to myeloma-induced osteolysis, and this phenomenon is believed to be induced by IL-17A, which was found to be significantly increased in MM patients [57]. Thus, targeting the IL-17 pathway might be a strategy to avoid osteoclagenesis, which is typical of this disease.

Under homeostatic conditions, stimulated pDCs are known to have a strong antigen-presenting potential. However, researchers already showed that in the MM setting these pDCs are defective in antigen presentation, leading to weak stimulation of T-cell proliferation [58]. For instance, in a study conducted by Chauhan et al., they found that there was an increase in the number of pDCs in the BM of myeloma patients. In addition, these cells interact directly with neoplastic plasma cells and produce soluble factors capable of promoting tumor growth and conferring drug resistance, including IL-3, IL-6, IL-10, IL-8, IL-15, VEGF, MCP-1, or CXCL12, which may allow pDCs survival, migration, and homing to BM. Importantly, by blocking either IL-3 or CXCL12, myeloma cell growth was markedly abrogated. In that study, although pDCs were resistant to anti-myeloma therapies, including bortezomib, lenalidomide, and dexamethasone, targeting TLRs with CpG oligodeoxynucleotides restored pDCs immune function and aborted pDCs-induced myeloma cell growth [59]. These evidences suggest that targeting pDCs-MM interaction might be an interesting therapeutic strategy to overcome drug resistance in MM. Similar to these findings, Ratta et al. deeply characterized circulating DCs isolated from MM patients, in which they found that the total amount of both mDCs and pDCs was significantly less in myeloma patients compared to healthy individuals. Interestingly, IL-6 production inhibited the growth of CD34+DC progenitors and switched their commitment into monocytic cells with phagocytic activity, abrogating antigen presentation ability. Still, blocking IL-6 using neutralizing antibodies did not completely revert this inhibitory effect [60], indicating that other soluble factors may contribute to DCs dysfunction, such as IL-10 or TGF-β [53,61,62]. Overall, these results suggest that one of the mechanisms by which neoplastic plasma cells evade immunity is only partially mediated by IL-6 production.

Conversely, in another study, Leone et al. found that both mDCs and pDCs accumulate in the BM of patients during MGUS-to-MM progression and these numbers correlate positively with the proportion of plasma cells in both MGUS and MM patients, indicating that DCs accumulation is proportional to tumor burden. Importantly, they seem to play a dual role in the MM BM microenvironment: on the one hand, DCs activate cytotoxic CD8+T cells against tumor cells through engulfment of apoptotic plasma cells and, on the other hand, DCs protect myeloma cells against CD8+T cell killing by downregulation of proteasome subunits in a contact-dependent manner involving the CD28-CD86/CD80 axis [63,64]. Thus, it is imperative to take this mechanism into account when designing novel immunotherapy strategies that aim to improve immune surveillance in the early stages of disease and/or break down immune defenses in active MM. For instance, blocking CD28 interactions using a CTLA-4 monoclonal antibody (mAb) could prevent the immune escape of myeloma cells and make them more susceptible to CD8+ T cells.

More recently, Ray et al. performed RNA sequencing analysis, where they identified the main pDCs-MM contact-dependent alterations responsible for tumor proliferation and immunosuppression. They found that the co-culture of pDCs and myeloma cells leads to an increase in the expression of CD73, TLR7/9, HDAC6, PD-L1, or IL3Rα/CD123 and reduces CASP3, BAK1, ADAM33, and BAD gene expression in tumor cells. Notably, by blocking CD73 it was possible to reactivate CD8+ T cell activity against myeloma cells. Moreover, the combination of an anti-CD73 with a TLR7 agonist increased even more the cytotoxic activity of lymphocytes. As such, these results bring novel therapeutic targets that might be used in order to improve MM therapy [65]. 

Altogether, these findings explain how the crosstalk between myeloma cells and DCs, either through cell-to-cell contact or soluble factors, impairs an effective anti-tumor immune response in the BM microenvironment, turning DCs into faithful allies of myeloma plasma cells. The understanding of the molecular interactions between myeloma and DCs may be turned into knowledge applied to the design of treatment approaches to MM. 

### 2.4. T and NK Cells

T- and NK-cell immunity plays a pivotal role in the interplay with MM plasma cells within the BM milieu. Defects in T cell distribution and function have been documented in MM, including the decrease of CD8+ and CD4+T cell frequency, abnormal Th1/Th2 ratio and impaired T cell responses [66]. Even in the precursor stages of plasma cell dyscrasia, evidence of T-cell dysfunction has been reported. For instance, MGUS patients showed increased levels of T cell exhaustion and a higher presence of regulatory T cells (Tregs) [67]. In SMM patients, there is a reduced expression of activation markers compared with healthy controls, such as CD25, CD28, and CD54 [68]. These phenotypic aberrations at early stages worsen throughout the disease course [69]. Although anti-myeloma effector responses seem to be efficiently primed, they subsequently fail as myeloma cells are able to circumvent T-cell effector functions through an array of mechanisms, such as defects in cytokine secretion, loss of proliferative capacity, impaired cytotoxicity, altered activity of transcription factors such as T-bet or the expression of immune checkpoint inhibitors (ICIs), including PD-1, CTLA-4, or TIGIT [70]. Immune checkpoint signaling is a key pathway to regulate the balance between activation and tolerance that, if altered, ultimately leads to the escape from immune surveillance. In MM patients, PD-1 is upregulated on T cells after activation [71] and direct interaction with its ligand (PD-L1) expressed in myeloma cells inhibits the T cell function by impairing proliferation and cytokine secretion [72]. In animal models, it was shown that the expression of exhaustion and senescence markers by T cells after autologous transplant is associated with clinical relapse, and treatment with anti-PD-1 ex vivo reinvigorated them to produce effector cytokines [73]. A higher number of PD-1+ T cells was detected in BM from relapsed/refractory (RR) MM patients than in newly diagnosed or partial/complete remission MM patients, and were associated with higher tumor burden and worst prognosis [74]. As PD-1 expression in T cells and PD-L1 in MM cells contribute to relapse and drug resistance mechanisms, immune-based therapeutic strategies that target checkpoint signaling with anti-PD-1 mAb could inhibit tumor cell growth and restore immune function [75]. Another co-inhibitory receptor, TIGIT was found to be expressed more frequently in CD8+T cells from MM patients than other ICIs, and those effector cells displayed limited cytokine responses. In Vk*MYC TIGIT-null mice, myeloma growth was delayed, and in wild type mice, tumor burden was reduced with anti-TIGIT treatment [76]. In a murine model of MM relapse after stem cell transplant, mice showed effector CD8+T cells with exhausted features, namely high TIGIT expression and low CD226 expression, and the anti-TIGIT treatment was able to improve disease control rates [21]. Nevertheless, a dysregulated BM immune microenvironment, with dysfunction of cytotoxic CD8+T cells or decreased levels of stem-like/resident memory T cells can lead to this type of therapeutic failure, compromising the efficacy of ICIs [77]. 

Immunomodulatory drugs currently used in MM treatment have demonstrated the ability to increase T-cell anti-tumor activity by promoting apoptosis and downregulating cytokine production [78]. As previously mentioned, within the MM BM niche, Th cells are also dysfunctional. Impaired proliferation of CD4+T cells, alterations in the balance between Th1 and Th2, with reduced Th1 cytokine production, overexpression of Th2 cytokines and expansion of pro-inflammatory Th17 cells, are some examples of how myeloma plasma cells contribute to immune response suppression and escape from T-cell surveillance [79]. Tregs, which can modulate overall immune responses against tumor cells, also have an important role in immune suppression and evasion of myeloma plasma cells [80]. Tregs inhibit the function of Th1 and Th17 cells, macrophages and DCs by direct cellular interaction or by secreting suppressive cytokines, such as IL-10 or TGF-β [81]. The frequency of Tregs in the BM microenvironment has been shown to be increased in MGUS patients that progress to MM, contributing to the early establishment of MM-related immunosuppression [82]. Furthermore, the expansion of Tregs has a negative impact on survival, which may hint at a potential therapeutic target [83]. Immunomodulatory treatments, such as lenalidomide and pomalidomide exert an anti-MM activity by inhibiting the proliferation and function of Tregs [84]. Similar to other immunosuppressive cells, such as MDSCs or Bregs, Tregs express high levels of CD38 and can be directly targeted with anti-CD38 antibodies to regulate the immune compartment and restore anti-MM T cell responses in the BM [85]. The therapeutic implications of Tregs are numerous, including in the development of vaccination strategies, as shown in a study in early-stage myeloma, where immune response failure was associated with the increase in Tregs frequency [86]. 

A small fraction of T cells, γδ T cells, were shown to have both adaptive and innate anti-myeloma effector functions [87]. γδ T cells produce IFN-γ and exhibit cytotoxic activity against MM cells after in vitro and in vivo activation with bisphosphonates and IL-2 [88,89]. Bisphosphonates, including pamidronate and zolendronic acid used in MM treatment, also enhance signaling through the receptor NKG2D, present not only on γδ T cells but also in CD8+T cells and NK cells, following engagement with the ligand major histocompatibility complex class-I related chain molecule A (MICA). MICA expression on the surface of plasma cells correlates inversely with disease stage, being higher in MGUS than in MM patients [90]. The shedding of MICA from myeloma cells leads to its increase in the PB, subsequently triggering a downmodulation of NKG2D, which suggests a possible immune escape process [90]. Though the use of γδ T cells as promising approaches for immunotherapy in MM could be hampered by the need to previously expand their number, the synergy with NK cells could be explored, as these cells seem to have similar cytotoxic mechanisms. 

NK cells are key innate immune players with direct cytotoxic activity and antibody dependent cellular cytotoxicity (ADCC), triggered upon the recognition of ligands on MM cells by activating receptors such as NKG2D, CD16, 2B4, NKp80 or DNAM-1 [91,92], regulated by major histocompatibility complex class I (MHC-I)-binding inhibitory receptors, such as killer cell Ig-like receptors (KIR) or CD94/NKG2A [93]. Contrary to CD8+T cells, NK cells respond to germline-markers of oncogenic transformation that are present on the surface of neoplastic plasma cells and not neoantigens presented by MHC-I, which is an advantageous characteristic in a low neoantigen presentation tumor, such as MM [94]. Myeloma cells can evade NK cell activity by maintaining human leukocyte antigen (HLA) expression [95] and by shedding MICA, leading to the downregulation or blocking of the NKG2D receptor on NK cells [96]. Activation of Tregs by MM cells has also been implicated in the inhibition of normal cytotoxic activity of NK cells [97]. Decrease in surface receptors as NKG2D or natural cytotoxicity receptors (NCRs) in BM NK cells, DNAM-1 in PB NK cells, and 2B4 in both PB and BM NK cells might contribute to the reduced functionality observed in MM patients [92,98]. NK cells from myeloma patients are usually dysfunctional compared to normal individuals and even to MGUS patients [99], and those with lower NK cytotoxicity present worse disease-free survival [100]. Alterations in the distribution of NK cell subset in MGUS and MM patients have also been detected, although there are conflicting results in this regard, possibly due to differences in the methodologies used [101,102,103]. Consequently, understanding the quantitative and functional alterations in NK cells of MM patients is increasingly relevant as new NK cell-based immunotherapies are being developed.

Overall, given all the strategies that myeloma cells use to escape lymphocytic surveillance in the BM niche, the development and optimization of efficient T and NK cell therapies are critically needed to improve outcomes in MM patients.

### 2.5. Regulatory B Cells

The role of B cell subsets in the MM BM microenvironment is still poorly described, however regulatory B cells (Bregs) have attracted attention as emerging prominent players in the initial stages of MM progression [104,105]. Bregs produce high amounts of IL-10 and TGF-β and can transform naive Th cells into CD4+CD25+FoxP3+ Tregs in an IL-10/TGF-β-dependent manner [106]. Generally, Bregs are described to maintain immune tolerance, suppress autoimmune and inflammatory responses as well as to suppress immune response during cancer progression. 

Neoplastic cells may attract naive B cells into the tumor microenvironment through chemoattractants and promote their differentiation into Bregs either directly, by releasing soluble factors or through cell contact, or indirectly, by recruiting other immune cells, such as IL-21-producing T cells, which in turn induce the local generation of microenvironment suppressive Bregs. In addition, this population may inhibit the ability of CD4+ and CD8+T cells to eliminate tumors [107,108], by inhibiting NK cell responses [109], causing Tregs proliferation [110,111], promoting the activity of TAMs and MDSCs, and directly promoting tumorigenesis and angiogenesis [112]. 

The work from Zhang and colleagues demonstrated that the frequency of Bregs in the BM of MM patients was significantly greater than in the PB, showing preferential accumulation of Bregs in the BM niche. Furthermore, they showed the Bregs’ dependency on MM cells for survival, through the depletion of CD138+ MM cells from the BM mononuclear cell culture with elotuzumab causing Bregs cells’ apoptosis. MM-derived BM Bregs were more immunosuppressive than their PB counterparts and were able to suppress anti-MM cell ADCC by NK cells [104], supporting Bregs as a novel cellular target of future therapeutics. 

In another study, Tai et al. have shown that Bregs highly express TACI compared with naive B cells in MM patients. In vitro studies have shown that APRIL is able to increase the frequency of BM Bregs and enhance IL-10 production leading to MM cells survival [113]. Moreover, the work from Zou and colleagues has shown an increase of Bregs in samples from MM patients and bortezomib was able to eliminate Bregs [105]. Despite the evidenced involvement of Bregs in MM, there have not been any in vivo studies published so far. 

This knowledge has been integrated to develop immunotherapies against MM. Table 1 describes novel and encouraging immunotherapeutic approaches against BM immunome in pre-clinical tests using MM mouse models. It should be noted that some aspects of immune deregulation in MM may be related with autoimmunity and response to infections. Indeed, several research groups have shown that sporadic MGUS and MM cases start by chronic antigen stimulation [114,115,116].

## 3. Conclusions

The present review considered the role of the BM immune microenvironment during MM progression when, undeniably, immune cells become myeloma’s best friends. As previously described, this deregulation happens through several mechanisms, by which MM cells use both myeloid and lymphoid populations to their advantage. Although several gaps in the knowledge of how myeloma relates to immune cells and vice-versa subsist, it is evident that, in order to recover the immune system, we need to break up this friendship and re-shape the MM BM immune niche. As such, novel immunotherapeutic approaches have been developed over the past years improving MM patients’ outcome. However, this remains an incurable hematological cancer and the search for the best combination treatment continues. 

## Figures and Tables

**Table 1:** In vivo pre-clinical tests showing promising molecules against multiple myeloma (MM) bone marrow (BM) milieu to overcome immunosuppression.

Molecular Target	In Vivo Pre-Clinical Studies	Reference
IL-18 in MDSCs	Long-term blockade of IL-18 delayed MM progression. Additionally, the combination of IL-18 mAb+Bortezomib significantly prolonged survival in MM models originally established as Bortezomib resistant.	Nakamura et al., 2018 [117]
piRNA-823	Silencing piRNA-823 in MM reduced the stemness of myeloma stem cells maintained by PMN-MDSCs, decreased tumour burden and angiogenesis in vivo.	Ai et al., 2019 [118]
DCs vaccination + Lenalidomide + anti-PD-1 mAb	This triple combination synergistically induced a stronger anti-tumour immune response by inhibiting MM growth in a murine model.	Vo et al., 2018 [119]
TRL9 agonist C792	C792 recovers pDCs ability to stimulate T cells and inhibits myeloma cell growth. Importantly, this cytotoxic activity enhances bortezomib, lenalidomide, SAHA or melphalan.	Ray et al., 2014 [120]
Clodronate-liposomes	Depletion of CD169+ bone marrow–resident macrophages in vivo abrogates myeloma growth.	Opperman et al., 2019 [121]
CD40 mAb + CpG (TLR9)	Macrophage-activating immunotherapy using CD40 plus CpG promoted anti-tumor effect in a RR MM murine model. This effect was increased when Tpl2 kinase was also inhibited showing an increase in both progression-free survival and overall survival.	Jensen et al., 2015 [122]
Anti-PD-1 mAb	Treatment with anti-PD-1 ex vivo reinvigorated T cells that expressed exhausted and senescence markers to produce effector cytokines.	Chung et al., 2016 [73]
Anti-TIGIT mAb expressed in T cells	In Vk*MYC TIGIT-null mice, myeloma growth was delayed and in wild type mice tumor burden was reduced with anti-TIGIT treatment.	Guillerey et al., 2018 [76]
CS1-NKG2D bi-specific antibody in NK cells	Anti-TIGIT treatment was able to improve disease control rates in a murine model of MM relapse after stem cell transplant. Prolonged survival in a humanized MM model.	Minnie et al., 2018 [21] Chang et al., 2018 [123]
